# FATIGABILITY, FITNESS, AND CHRONO-NUTRITION: RESULTS FROM THE STUDY OF MUSCLE, MOBILITY AND AGING (SOMMA)

**DOI:** 10.1093/geroni/igad104.1253

**Published:** 2023-12-21

**Authors:** Nancy Glynn, Stephen Kritchevsky, Jennifer Schrack

## Abstract

The Study of Muscle, Mobility and Aging (SOMMA) is a new longitudinal cohort study conducted at the University of Pittsburgh and Wake Forest University School of Medicine, coordinated by the San Francisco Coordinating Center, with management of biological specimens by the Translational Research Institute at AdventHealth. SOMMA aims to provide the foundation for discoveries in the biology of human aging, mobility and other age-related phenotypes. SOMMA enrolled 879 older women and men; mean age 76.3±5.0 years (range 70-94); mean walking speed 1.04±0.20 m/s; 59.2% women, and 15.8% identify as other than Non-Hispanic White. Our symposium will introduce novel baseline SOMMA findings on key measures related to fatigability, fitness, and chrono-nutrition. First, Dr. Schumacher will share results on the association between perceived physical and mental fatigability and cognitive function. Next, to begin to decipher how skeletal muscle energetics impact aging phenotypes, Ms. Gay will examine the relation between skeletal muscle energetics (ATPmax and maximal complex I&II supported oxidative phosphorylation [maxOXPHOS]) and age-related physical fatigability. Our next two presentations will focus on cardiorespiratory fitness (VO2peak) measured by gold standard treadmill cardiopulmonary exercise testing (CPET), which declines with age. Ms. Moffit will explore whether a usual-paced 400m walk can predict VO2peak, and Dr. Erickson will examine the association between CPET measured VO2peak and circadian functions derived from rest-activity-rhythms extracted from wrist-worn accelerometers. Lastly, Dr. Farsijani will explore chrono-nutrition behaviors and their association with muscle health (i.e., D3-creatine, leg power). Discussant, Dr. Jennifer Schrack will provide insight to SOMMA’s novel work.

